# Autogenous grafts versus substitute scaffolds for soft tissue augmentation in implant therapy: A systematic review

**DOI:** 10.1002/jper.70104

**Published:** 2026-09-14

**Authors:** Gustavo Avila‐Ortiz, Sandra Stuhr, Miha Pirc, Emilio Couso‐Queiruga, Franz J. Strauss, Leandro Chambrone, Daniel Thoma, Ronald Jung

**Affiliations:** ^1^ Department of Periodontics and Oral Medicine University of Michigan School of Dentistry Ann Arbor Michigan USA; ^2^ Clinic of Reconstructive Dentistry University of Zurich Zurich Switzerland; ^3^ Department of Oral Surgery and Stomatology University of Bern School of Dental Medicine Bern Switzerland; ^4^ Universidad Autonoma de Chile Temuco Chile; ^5^ Evidence‐Based Hub Interdisciplinary Research Center Egas Moniz (CiiEM) Egas Moniz‐High Education Cooperative, Almada Caparica Portugal; ^6^ Unit of Basic Oral Investigations School of Dentistry Universidad El Bosque Bogotá Colombia

**Keywords:** dental implants, esthetics, grafts, oral health, patient‐reported outcome measures, plastic surgery, tissue scaffolds

## Abstract

**Background:**

To analyze the effect of autogenous soft tissue grafts or substitute scaffolds on the outcomes of soft tissue augmentation (STA) performed in the context of implant therapy.

**Methods:**

The protocol of this PRISMA 2020‐compliant systematic review was registered in PROSPERO (CRD42024611477). Articles reporting the findings of randomized controlled trials (RCTs) that met the eligibility criteria were identified through a literature search. Following final article selection, data were extracted, including relevant clinician‐ and patient‐reported outcomes and experiences (CROs and PROs/PREs). Risk of bias assessment was performed using the RoB2 tool. Network meta‐analyses (NMA) were performed when feasible to compare the performance of different techniques.

**Results:**

Forty RCTs were selected. Risk of bias assessment revealed that the methodological quality of the selected studies was generally high. Although CROs and PROs/PREs were inconsistently reported, it was possible to conduct five sets of NMA regarding mucosal thickness (MT) and keratinized mucosa width (KMW) changes. These analyses revealed that the use of an autogenous connective tissue graft in a bilaminar approach is the best treatment for MT augmentation regardless of the timing, while the application of a free mucosal graft over a partial‐thickness vascular bed is generally the best to obtain KMW gains. Alternatively, other treatment options involving the use of specific types of substitute scaffolds were considered as viable alternatives given their similar performance, particularly in terms of MT gain. Additionally, it was found that, compared with autogenous soft tissue grafts, the use of substitute scaffolds is consistently associated with lower postoperative discomfort.

**Conclusions:**

Autogenous soft tissue grafts should be considered as the primary choice to achieve predictable MT and KMW gains in the context of implant therapy. The use of specific substitute scaffolds may render similar STA outcomes with lower postoperative morbidity.

## Plain language summary

Thick and firm gum tissue around dental implants is important to support long‐term health, comfort, and esthetics. This review was aimed at analyzing the effectiveness of two main approaches for increasing gum tissue around dental implants: using a patient's own tissue (autogenous grafts) or using substitute materials (scaffolds made from donated or engineered sources). The authors analyzed 40 clinical trials from different countries that compared these methods. The review found that connective tissue grafts from the patient remain the most reliable way to increase gum thickness around implants. Free mucosal grafts are most effective for widening the band of keratinized gum. However, substitute scaffolds such as collagen matrices and dermal grafts can achieve similar results in some situations and are consistently associated with less pain and discomfort after surgery. Overall, autogenous grafts should be considered the first choice when predictable outcomes are essential, but scaffolds are a valuable alternative that reduce patient morbidity. More long‐term research is needed to test novel substitute materials and techniques, and the stability of results over time.

## 1 INTRODUCTION

Soft tissue augmentation (STA) has become an integral component of contemporary implant therapy. STA is primarily indicated to enhance the phenotypical features of the peri‐implant soft tissue with the purpose of achieving satisfactory and long‐lasting clinician‐ and patient‐reported outcomes and experiences (CROs and PROs/PREs) related to health, function, and esthetics.^1^, ^2^ The peri‐implant soft tissue phenotype may be defined as the morphologic and dimensional features characterizing the clinical presentation of the soft tissues that surround osseointegrated implants. According to current evidence, the essential components of the peri‐implant soft tissue phenotype are the keratinized mucosa width (KMW), the mucosal thickness (MT), and the supracrestal tissue height (STH).^3^ The characteristics of these three phenotypical constituents on the crestal region of the ridge are particularly relevant given the impact that they may have on esthetic integration, patient comfort, and the prevention of biological complications, including diseases, namely peri‐implant mucositis and peri‐implantitis,^4^ or conditions such as peri‐implant soft tissue dehiscences (PSTDs).^5^, ^6^, ^7^

Surgical approaches for STA typically involve the use of replaced or displaced flaps (e.g., envelope or tunnel) in conjunction with an autogenous graft or a substitute scaffold.^8^, ^9^ While autogenous grafts are portions of living tissue harvested from the host to repair a defect or to compensate for a deficiency, substitute scaffolds are exogenous materials of biologic (e.g., xenogeneic or allogeneic) or synthetic origin that do not contain native cells.^10^ Each has distinct advantages and limitations.

Autogenous soft tissue grafts, including free mucosal grafts (FMG) and connective tissue grafts (CTG), are generally considered the gold standard due to their unsurpassable biocompatibility, biological properties, and predictable CROs when employed in periodontal and implant‐related plastic surgery.^2^, ^11^ However, the use of autogenous grafts is associated with drawbacks such as increased risk of intra‐ and post‐operative complications (e.g., hemorrhage), longer surgical times, and donor site morbidity (e.g., pain and discomfort).^12^

To address these downsides, substitute scaffolds such as xenogeneic collagen matrices (XCM) and allogeneic dermal matrices (ADM) have been developed, investigated, and widely utilized in clinical practice over the past two decades.^13^, ^14^ The use of these alternative biomaterials reduces surgical invasiveness, patient morbidity, and operating time by eliminating the need to harvest tissue from an intraoral donor site (often the palatal mucosa), with some studies reporting similar or even superior outcomes compared with autogenous grafts, particularly in terms of PROs.^15^, ^16^, ^17^ However, their efficacy in achieving predictable and stable peri‐implant soft tissue gains compared with autogenous soft tissue grafts, particularly in the long‐term, remains debated, mainly due to heterogeneity and limited follow‐up periods within the highest‐level available evidence.^9^, ^18^, ^19^

A comprehensive understanding of the comparative efficacy of different STA approaches, implying the use of autogenous grafts or substitutes, is essential for clinicians to make evidence‐based decisions and to tailor treatment plans to individual patient needs. Additionally, in the continuum of care of implant therapy, STA may be performed prior to, at the time of (i.e., immediate or delayed placement protocols), or at any time point after implant placement, including implant uncovering or after delivery of a provisional or final implant‐supported restoration, depending on site‐specific variables (e.g., phenotypical features), treatment goals, and patient's preferences. These considerations may add multiple layers of complexity to clinical decision‐making processes.

Hence, considering these important procedural aspects, as well as the introduction of novel materials, technical developments, and the increase of available evidence on this topic published in recent years,^20^ the conduction of a new systematic review (SR) is justified. The primary aim of this systematic review, which was commissioned to inform the 2025 Consensus of the American Academy of Periodontology (AAP), Osteology Foundation (OF) and the Sepa Foundation (SEPA) on “New Technologies in Oral Reconstructive Medicine” was to analyze the effect of using autogenous soft tissue grafts or substitute scaffolds for STA according to a panel of relevant CROs and PROs/PREs, considering the timing of augmentation (i.e., at the time of or after implant placement) and implant placement modality (i.e., immediate or delayed).

The following focused question was addressed: “In partially edentulous adult patients presenting mucosal deficiencies, what is the performance of different modalities of STA at the time of or following immediate or delayed implant placement in terms of peri‐implant soft tissue gains and other relevant CROs and PROs?”.

## 2 MATERIALS AND METHODS

The protocol of this review was registered in the International Prospective Register of Systematic Reviews (PROSPERO) with the identification code CRD42024611477. This review adheres to the guidelines of the Preferred Reporting Items of Systematic Reviews and Meta‐Analyses (PRISMA) 2020 statement.^21^

The following PICO framework was designed to address the review focused question:

**PICOS outline**

- POPULATION:
  Adult patients presenting structural deformities that underwent surgical interventions for STA at the time of or after implant placement.
- INTERVENTION:
  Any type of surgical intervention for STA involving the use of either autogenous grafts or substitute scaffolds (i.e., allogeneic or xenogeneic materials) with or without the use of biologics.
- COMPARISON:
  Any type of comparisons among interventions of interest or with any control treatments.
- OUTCOMES:
  Based on the Implant Dentistry‐Core Outcomes Sets and Measures—ID‐COSM,^20^, ^22^ the following outcomes of interest were selected:
  CROs
  − Clinical: Linear changes in peri‐implant KMW, MT or STH (assessed digitally or clinically), linear changes in marginal mucosa position (assessed digitally or clinically), changes in peri‐implant mucosa volume (assessed digitally), implant survival rate, incidence of peri‐implant mucositis and peri‐implantitis.
  − Radiographic: Marginal bone level changes (assessed digitally).
  − Esthetic: Assessment of esthetics using a standardized method (e.g., Pink Esthetic Score [PES]).
  − Safety: Occurrence of postoperative complications and adverse events.
  PROs/PREs
  − Overall satisfaction or perceived benefit using questionnaires, visual analog scales (VAS) or numeric rating scales (NRS).
  − Esthetic perception using questionnaires, VAS, or NRS.
  − Morbidity: Perceived postoperative pain or discomfort using a VAS or NRS, consumption of analgesics or painkillers.
- STUDY DESIGN:
  Randomized Controlled Trials (RCTs).

### 2.1 Criteria for inclusion

#### 2.1.1 Type of study, follow‐up, and participants

Articles reporting RCTs were considered eligible if the following criteria were met:

- Surgical treatment of adult patients (aged ≥18 years).
- Simultaneous or delayed STA following immediate or delayed implant placement regardless of anatomical location (anterior or posterior).
- A follow‐up period of at least 6 months.
- At least 10 participants per treatment arm and/or less than a 20% dropout rate at the short‐term (6–12 months) follow‐up.

#### 2.1.2 Type of intervention of interest

Surgical interventions primarily aimed at KMW, MT and/or STH augmentation using either an autogenous soft tissue graft (i.e., FMG or CTG) or a substitute (i.e., allogeneic or xenogeneic) with or without biologics.

Studies reporting on variations of the same technical intervention (e.g., open flap with vs. without vertical releasing incisions, open flap vs. tunnel flap, etc.) were included in the review.

### 2.2 Information sources and search strategy

Three electronic databases were searched, namely National Library of Medicine (MEDLINE/PubMed), Cochrane Central Register of Controlled Trials (CENTRAL), and EMBASE, according to specific strategies developed for each database, based on the following for MEDLINE/PubMed:

1. Gingival graft OR connective tissue graft OR free gingival graft OR acellular dermal matrix OR dermal matrix allograft OR collagen matrix OR xenogeneic collagen matrix.
2. Autologous hemoderivatives OR platelet rich fibrin OR platelet‐rich fibrin OR leukocyte platelet rich fibrin OR platelet rich plasma OR enamel matrix derivatives OR EMD OR growth factors OR recombinant human platelet‐derived growth factor BB OR rhPDGF‐BB.
3. Soft‐tissue augmentation OR keratinized tissue OR tissue thickness OR soft tissue thickness OR supracrestal soft tissue height augmentation OR keratinized peri‐implant mucosa augmentation OR peri‐implant STA.
4. #1 OR #2.
5. #3 AND #4.
6. Implant OR dental implant OR dental, implants OR dental implantation OR peri‐implant.
7. #5 AND #6.

The electronic search aimed at identifying articles published in English language between January 1, 2000, and November 1, 2024. Additionally, the references included in other SRs on this topic published between September 30, 2019 and September 30, 2024 were searched.^1^, ^2^, ^8^, ^9^, ^13^, ^14^, ^15^, ^18^, ^23^, ^24^ Per agreement with the consensus leadership, the grey literature was not searched.

### 2.3 Selection process

Two reviewers (M.P. and F.J.S.) independently read the title and abstract of the entries obtained from the literature search. Inter‐examiner calibration was achieved by open discussion and comparison after independent assessment of the first 200 records. After completing the screening process, both reviewers read individually through the full‐text version of the potentially eligible studies. Final article selection was dictated by the eligibility criteria (see Section 2.1). For a series of studies on the same population, only the article reporting the longest follow‐up was selected. When disagreement regarding the inclusion of a specific article occurred, both reviewers had an open discussion. If no agreement was achieved, another co‐author (D.T.) made the final decision. Following article selection, Cohen's kappa coefficient (*k*) was calculated to determine the degree of inter‐examiner agreement.

### 2.4 Data extraction

Data extraction was preliminarily performed by two independent examiners (S.S. and E.C‐Q.). Examiners were calibrated by using a random selection of five articles to ensure consistency in the data extraction process and the terminology employed. Final data accuracy and consistency were independently verified by a third author (G.A‐O.). Any missing information that could contribute to this review was requested from the corresponding author(s) via email communication.

### 2.5 Methodological quality and risk of bias assessment

The assessment of methodological quality and risk of bias of each included RCT was performed by two independent reviewers (M.P. and F.J.S.) using version 2 of the Cochrane risk‐of‐bias tool for RCTs (RoB2).^25^ RoB2 individual domains assess the following types of bias: (1) bias arising from the randomization process; (2) bias due to deviations from intended interventions; (3) bias due to missing outcome data; (4) bias in measurement of the outcome; and (5) bias in the selection of the reported result.

Based on this assessment, the risk of bias of each included RCT was rated as:

1. Low risk of bias (plausible bias unlikely to seriously alter the results) if all domains were at low risk of bias;
2. Unclear risk of bias (plausible bias that raises some doubt about the results) if ≥1 domains were at risk unclear bias;
3. Risk of bias, but not to be at high risk of bias for any domain; or
4. High risk of bias (plausible bias that seriously weakens confidence in the results) if ≥1 domains were at high risk of bias, or the study is judged to have unclear risk of bias for multiple domains in a way that substantially lowers confidence in the result.

### 2.6 Data extraction

Extracted data were organized into evidence tables. In addition to the reported outcomes of interest, Supporting Information included year of publication and author(s), country(ies) and setting(s) in which the study was conducted, study design, initial and final number of participants, sex and age distribution, and description of intervention(s).

### 2.7 Data synthesis

Frequentist network meta‐analyses (NMA) and associated statistical calculations were performed using MetaInsight v6.2.0, a web‐based tool for analyzing, interrogating, and visualizing NMA using R‐shiny and netmeta.^26^ Random effects models were applied to calculate the mean differences (MD) with respective 95% confidence intervals (CIs) for the analyzable outcomes of interest (i.e., MT and KMW changes). Analyses were conducted using the “longest” short‐term data available (i.e., between 6 and 12 months follow‐ups) reported in the included articles. Following a frequentist point estimation, relative treatment effects in ranked order for all studies were calculated, where treatments were ranked from “best to worst” (i.e., higher score meant better treatment). Global inconsistency in each model was calculated, and a *p*‐value < 0.05 for the difference between the residual deviance from an inconsistency model and the residual deviance from the NMA model was considered suggestive of significant inconsistency. Subgroup analyses or pairwise meta‐analyses were also performed, if applicable, to further explore the data. Forest plots of the pooled effect estimates, their associated uncertainty (95% CIs), and the global heterogeneity (as measured by the between study standard deviation values or “tau” for all interventions compared with the reference treatment) were calculated. For all analyses, *p*‐values < 0.05 indicated a significant difference between treatment approaches.

## 3 RESULTS

### 3.1 Article selection

**FIGURE 1:**
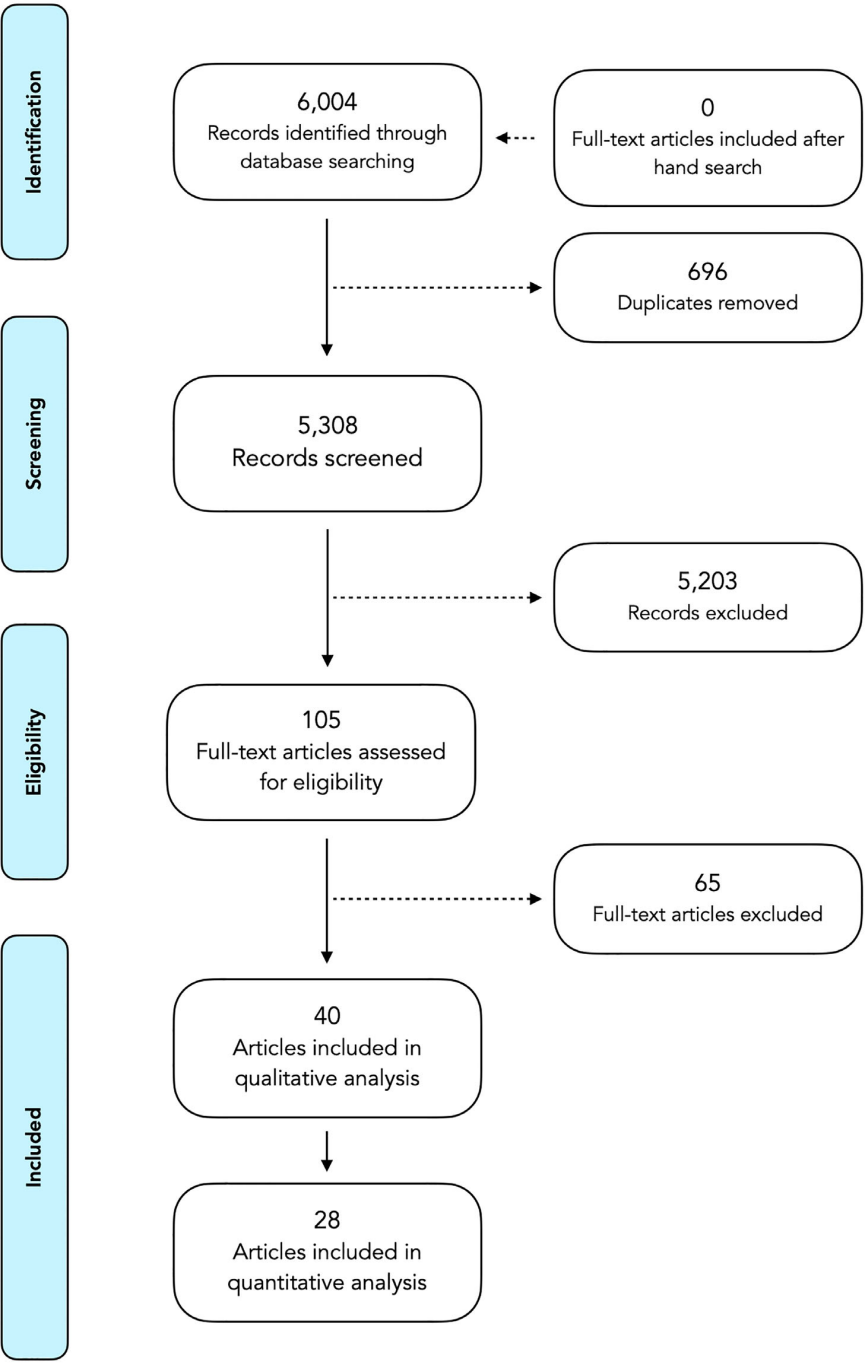
Flow chart depicting the article selection process.

The initial database search yielded a total of 6004 entries, of which 2647 were found in PubMed, 2266 in CENTRAL, and 1091 in EMBASE. No additional articles were identified through manual searching. Following removal of duplicates, 5308 entries remained. After title and abstract screening, 105 articles were selected for full‐text review. Sixty‐five of these articles were excluded after full‐text review. The list of excluded articles and reasons for exclusion is displayed in Table S1 (available in the online *Journal of Periodontology* under “Supporting Information”). Thus, 40 articles corresponding to 40 different clinical trials were finally selected.^27^, ^28^, ^29^, ^30^, ^31^, ^32^, ^33^, ^34^, ^35^, ^36^, ^37^, ^38^, ^39^, ^40^, ^41^, ^42^, ^43^, ^44^, ^45^, ^46^, ^47^, ^48^, ^49^, ^50^, ^51^, ^52^, ^53^, ^54^, ^55^, ^56^, ^57^, ^58^, ^59^, ^60^, ^61^, ^62^, ^63^, ^64^, ^65^, ^66^ A flowchart illustrating the article selection process is depicted in Figure 1. Inter‐examiner agreement kappa scores for title/abstract review and for full text review were 0.851 and 0.794, respectively, indicating a high level of agreement.

### 3.2 Study characteristics

Articles selected in this SR were published between 2010 and 2024. Five studies were conducted in the USA,^28^, ^34^, ^47^, ^60^, ^64^ four in India,^33^, ^37^, ^49^, ^50^ four in Italy,^29^, ^32^, ^36^, ^40^ three in Belgium,^31^, ^35^, ^58^ three in China,^43^, ^44^, ^53^ three in Egypt,^27^, ^38^, ^41^ three in the Netherlands,^62^, ^65^, ^66^ three in Spain,^48^, ^55^, ^56^ two in Germany,^42^, ^54^ two in Lithuania,^51^, ^52^ two in Switzerland,^57^, ^61^ one in Austria,^63^ one in Brazil,^46^ one in Portugal,^39^ one in Turkey,^30^ one in Russia,^59^ and one in Syria,^45^ reflecting a high diversity in terms of geographical location. The majority of studies (*n* = 28) were conducted in academic settings,^27^, ^28^, ^30^, ^31^, ^33^, ^35^, ^36^, ^38^, ^39^, ^40^, ^41^, ^44^, ^45^, ^46^, ^47^, ^50^, ^53^, ^54^, ^55^, ^56^, ^57^, ^59^, ^60^, ^61^, ^62^, ^64^, ^65^, ^66^ while four studies were executed in a private practice environment,^34^, ^51^, ^52^, ^63^ and two were conducted in both private practice and academic institutions.^29^, ^48^ In the rest, the setting was unclear. Of the 40 selected RCTs, only two had a split‐mouth design,^45^, ^63^ whereas the rest had a parallel‐arms design. A total of 1293 patients were enrolled in the RCTs included in this SR. Smokers were included in 19 studies,^27^, ^28^, ^30^, ^31^, ^34^, ^35^, ^39^, ^43^, ^45^, ^46^, ^49^, ^50^, ^58^, ^59^, ^62^, ^63^, ^64^, ^65^, ^66^ while they were excluded in only six trials.^29^, ^32^, ^47^, ^52^, ^57^, ^60^ In the rest of the cstudies, smoking status was not reported, or it was unclear.

The primary therapeutic purposes of the surgical interventions performed in the selected studies were MT augmentation (*n* = 27),^27^, ^28^, ^31^, ^32^, ^33^, ^34^, ^35^, ^36^, ^38^, ^39^, ^40^, ^41^, ^42^, ^44^, ^45^, ^46^, ^47^, ^50^, ^51^, ^55^, ^58^, ^61^, ^62^, ^63^, ^64^, ^65^, ^66^ KMW augmentation (*n* = 10),^29^, ^30^, ^37^, ^43^, ^48^, ^49^, ^53^, ^54^, ^56^, ^59^ STH augmentation (*n* = 2),^52^, ^57^ or correction of PSTDs (*n* = 1).^60^ A wide variety of surgical modalities of treatment were reported in these studies, involving full‐ or partial‐thickness approaches, and tunnel, envelope or trapezoidal flaps with or without displacement. With the primary objective of gaining KMW, the use of an autogenous free mucosal/gingival graft was described in six trials.^30^, ^37^, ^43^, ^53^, ^54^, ^59^ With the goal of augmenting MT and/or KMW autogenous CTGs were employed in 28 studies.^27^, ^29^, ^31^, ^32^, ^34^, ^35^, ^36^, ^38^, ^39^, ^40^, ^41^, ^44^, ^45^, ^46^, ^47^, ^48^, ^49^, ^50^, ^51^, ^55^, ^56^, ^61^, ^62^, ^63^, ^64^, ^65^, ^66^ Interestingly, STH augmentation was attempted with the use of ADM in two studies.^52^, ^57^ In this sense, the use of substitute scaffolds was reported in a total of 21 studies.^28^, ^29^, ^30^, ^32^, ^34^, ^37^, ^41^, ^43^, ^47^, ^48^, ^50^, ^51^, ^52^, ^53^, ^54^, ^56^, ^57^, ^58^, ^59^, ^61^, ^65^ More specifically, bilayer XCMs were tested in nine trials,^32^, ^37^, ^43^, ^48^, ^53^, ^54^, ^56^, ^59^, ^65^ volume‐stable XCMs in four,^34^, ^41^, ^58^, ^61^ xenogeneic dermal matrices in two,^29^, ^51^ and ADMs in six clinical investigations.^28^, ^30^, ^47^, ^50^, ^52^, ^57^ Only in one of these studies, was ADM left intentionally exposed to the oral cavity (i.e., uncovered by an overlying mucosal flap).^30^ Proper and direct assessments of the performance of an autogenous CTG compared with a substitute scaffold were conducted in 12 RCTs,^29^, ^32^, ^34^, ^41^, ^47^, ^48^, ^50^, ^51^, ^56^, ^58^, ^61^, ^65^ whereas only six comparative studies of FMG versus substitute scaffolds could be identified.^30^, ^37^, ^43^, ^53^, ^54^, ^59^ Biologics were tested in three RCTs.^33^, ^42^, ^45^ Notably, all of these biologics were hemoderivatives (i.e., platelet‐rich fibrin and concentrated growth factor graft). Only in one of these three trials was the performance of the biologic agent directly compared with an autogenous graft.^45^

In 24 studies STA was performed at the time of implant placement,^27^, ^28^, ^31^, ^33^, ^36^, ^38^, ^39^, ^40^, ^41^, ^42^, ^44^, ^46^, ^47^, ^50^, ^51^, ^52^, ^57^, ^58^, ^61^, ^62^, ^63^, ^64^, ^65^, ^66^ while in 15 studies it was executed at some time point after implant placement.^29^, ^30^, ^32^, ^34^, ^37^, ^43^, ^45^, ^48^, ^49^, ^53^, ^54^, ^55^, ^56^, ^59^, ^60^ In one study, STA was performed at the time of implant placement in the test group, whereas in the control group, it was done after implant placement.^35^ Regarding modality of implant placement, implants were placed according to a delayed protocol in 15 studies,^27^, ^28^, ^30^, ^31^, ^36^, ^38^, ^41^, ^45^, ^46^, ^51^, ^57^, ^58^, ^61^, ^63^, ^65^ immediate implant placement was performed in 10 investigations,^35^, ^39^, ^40^, ^44^, ^47^, ^50^, ^51^, ^62^, ^64^, ^66^ and this methodological aspect was not reported or it was unclear in the rest of the selected articles. The follow‐up time reported in the majority of selected studies was generally short (up to 12 months), ranging from 6 months to a maximum of 5 years, which was only reported in two studies.^61^, ^65^

For further and more specific details on the information extracted from the selected articles (e.g., study characteristics, participants, therapeutic approaches, outcomes, etc.), see the data collection form under online “Supporting Information” (Appendix 1 in the online *Journal of Periodontology*).

### 3.3 Risk of bias assessment

**FIGURE 2:**
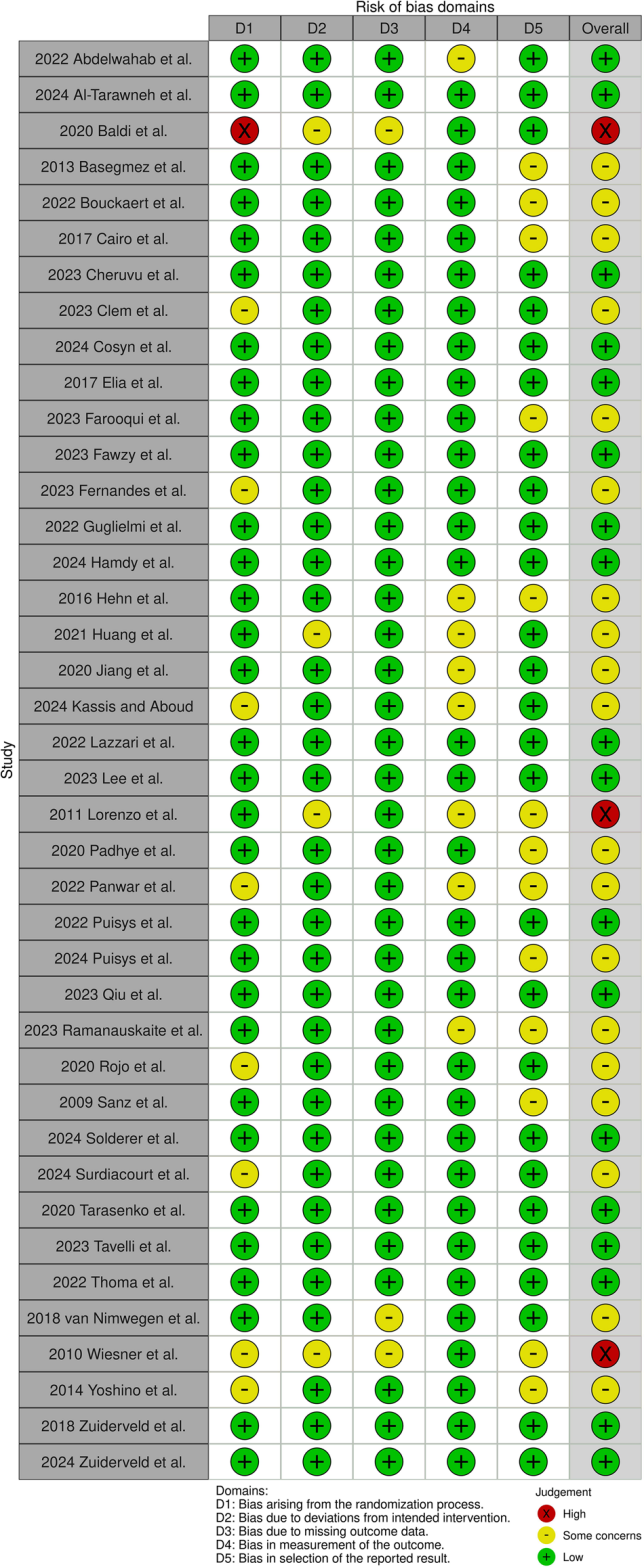
Risk of bias assessment for each selected randomized controlled trial.

**FIGURE 3:**
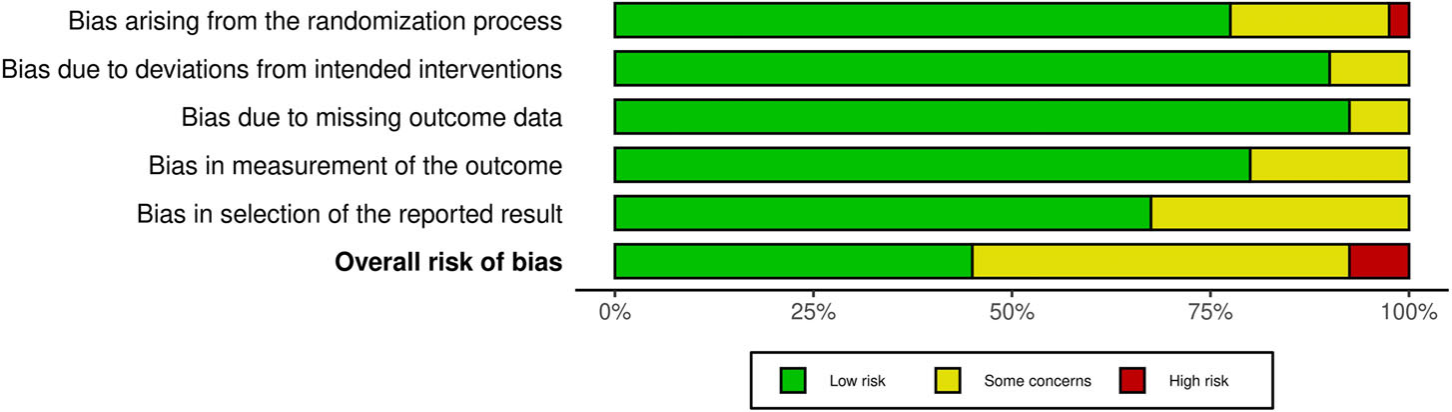
Risk of bias assessment per domain of the RoB2 tool.

The risk of bias assessment of each individual study included in this systematic review revealed that 18 of the trials had a low risk of bias,^27^, ^28^, ^33^, ^35^, ^36^, ^38^, ^40^, ^41^, ^46^, ^47^, ^51^, ^53^, ^57^, ^59^, ^60^, ^61^, ^65^, ^66^ while 19 of them were allocated in the category of unclear risk of bias, which indicates that some concerning methodological aspects were identified.^30^, ^31^, ^32^, ^37^, ^39^, ^42^, ^43^, ^44^, ^45^, ^49^, ^50^, ^52^, ^54^, ^55^, ^56^, ^58^, ^62^, ^64^ Only three studies exhibited a high risk of bias,^29^, ^48^, ^63^ as shown in Figure 2. A detailed assessment of the results pertaining to each domain of the RoB2 tool showed that a higher overall risk of bias was primarily associated to “bias in measurement of the outcome” and “bias in selection of the reported result”. This was largely due to the absence of a registered protocol, which hindered a comprehensive risk assessment (Figure 3).

### 3.4 Outcomes of interest: Qualitative assessment

#### 3.4.1 CROs

Outcomes of interest were inconsistently reported in the selected articles. Among the most relevant CROs pertaining to STA, MT changes was the most frequently reported (18 articles = 45%),^27^, ^28^, ^33^, ^34^, ^35^, ^40^, ^41^, ^43^, ^44^, ^46^, ^47^, ^50^, ^53^, ^58^, ^60^, ^61^, ^62^, ^63^ followed by KMW changes (12 articles = 30%),^29^, ^30^, ^33^, ^40^, ^43^, ^47^, ^50^, ^53^, ^54^, ^59^, ^60^, ^61^ marginal mucosa position changes (9 articles = 22.5%),^31^, ^36^, ^39^, ^47^, ^48^, ^58^, ^62^, ^64^, ^65^ soft tissue volume % change (3 articles = 7.5%),^28^, ^34^, ^39^ and STH changes (3 articles = 7.5%).^28^, ^52^, ^57^ An overall evaluation of these outcomes indicates that different STA approaches involving the use of autogenous grafts or substitute scaffolds generally result in clinical improvements from baseline dimensions compared with control therapies (e.g., displaced flap alone). However, the range of the effect appears to vary substantially across STA strategies.

Implant survival rate was reported in 20 articles (50%) with values that ranged between 78% and 100% with a follow‐up between 6 months and 5 years.^27^, ^28^, ^29^, ^31^, ^32^, ^38^, ^39^, ^40^, ^44^, ^47^, ^50^, ^51^, ^52^, ^57^, ^58^, ^62^, ^63^, ^64^, ^65^, ^66^ It should be highlighted that most implant survival rates reported were above 95%, except in three articles.^29^, ^38^, ^57^ Marginal bone level changes were reported in 15 articles (37.5%),^28^, ^29^, ^31^, ^35^, ^46^, ^47^, ^51^, ^52^, ^57^, ^58^, ^61^ ^63^, ^64^, ^65^, ^66^ with values that did not exceed a loss of 1.5 mm, except in the control group of one study.^29^ Clinician‐reported esthetic assessments were performed in 20 studies (50%) using different scoring systems,^27^, ^29^, ^31^, ^34^, ^37^, ^38^, ^41^, ^43^, ^46^, ^51^, ^53^, ^55^, ^58^, ^60^, ^61^, ^62^, ^63^, ^64^, ^65^, ^66^ but predominantly the PES.^67^ These outcomes generally indicate that, compared with substitute scaffolds, the use of an autogenous CTG was associated with slightly superior esthetic outcomes when the primary therapeutic goal is MT and volume gain. Conversely, in those studies focused on the performance of autogenous FMG compared with substitute scaffolds to achieve KMW gain, it was observed that the texture, color, and tissue integration tended to be superior when a substitute scaffold was employed.^37^, ^43^, ^53^ Incidence of peri‐implant mucositis and peri‐implantitis was only reported in the same three articles,^31^, ^58^, ^61^ with only two cases of peri‐implantitis diagnosed over a follow‐up period of 3 years within the test group in one study.^58^ According to the information reported in the selected literature, occurrence of postoperative complications was a rare event as they were only reported in 10 articles (25%) and mostly fell in the category of mild to moderate, including early implant failures, donor site bleeding or tissue necrosis, excessive edema, and premature wound dehiscence.^27^, ^28^, ^33^, ^36^, ^37^, ^38^, ^52^, ^58^, ^59^, ^64^

#### 3.4.2 PROs/PREs

Regarding PROs and PREs, three categories could be identified in the selected literature: (1) Overall satisfaction and perceived benefit; (2) perceived esthetics; and (3) perceived postoperative discomfort (morbidity).

Overall satisfaction and perceived benefit was assessed in 13 studies (32.5%),^27^, ^28^, ^29^, ^32^, ^34^, ^41^, ^43^, ^47^, ^61^, ^62^, ^63^, ^65^, ^66^ employing a variety of tools, some of which were standardized, such as OHIP‐14 and 10‐point VAS, while others were customized (i.e., questionnaires), which made it unfeasible to perform a quantitative analysis of these data. Nonetheless, a qualitative evaluation of this information indicates that patients who received STA were equally or slightly more satisfied with the overall experience compared with patients randomized to control groups.

Similarly, esthetic perceptions were reported in 11 articles (27.5%),^27^, ^29^, ^32^, ^34^, ^47^, ^53^, ^58^, ^60^, ^62^, ^63^, ^65^ which were assessed primarily through non‐standardized tools, such as questionnaires pertaining to pink and white esthetics, and VAS. Interestingly, a qualitative assessment of this information revealed that the esthetic perception of patients who underwent STA did not substantially differ between those who received an autogenous CTG or a substitute scaffold, with the exception of one study. In this investigation it was observed that 100% of the patients who had an autogenous CTG were fully satisfied from an esthetic perspective, while this was the case in 81.82% of those who received an ADM and in 78.57% of the patients in the control group (no STA).^47^ Conversely, in the only study that compared the performance of an FMG with a substitute scaffold (i.e., bilayer XCM) and patient‐reported esthetic perception was assessed with a 10‐point VAS, the mean score in the test (XCM) and control (FMG) group was 6.53 ± 0.71 and 4.13 ± 1.16, respectively, indicating that the esthetic results associated with the use of the substitute was superior.^53^

Perceived postoperative discomfort was reported in 15 articles (%).^32^, ^37^, ^39^, ^40^, ^41^, ^43^, ^46^, ^49^, ^53^, ^54^, ^56^, ^58^, ^59^, ^60^, ^61^ This outcome was assessed with a 10‐ or 100‐point VAS between 7 and 14 days in most studies. Although a quantitative analysis could not be performed due to high methodological variability and the lack of sufficient data, in those trials that compared the performance of autogenous grafts with substitute scaffolds,^32^, ^37^, ^41^, ^43^, ^53^, ^54^, ^56^, ^58^, ^59^, ^61^ it was consistently found that patients who underwent STA involving an autogenous graft, either a CTG or an FMG, experienced greater discomfort that progressively tapered down up to 30 days.

### 3.5 Outcomes of interest: Quantitative assessment

**FIGURE 4:**
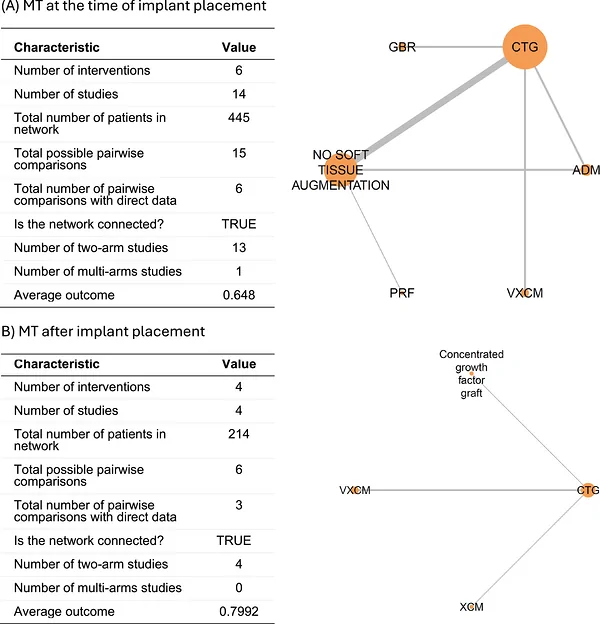
Network meta‐analysis plots for mucosal thickness changes.

**FIGURE 5:**
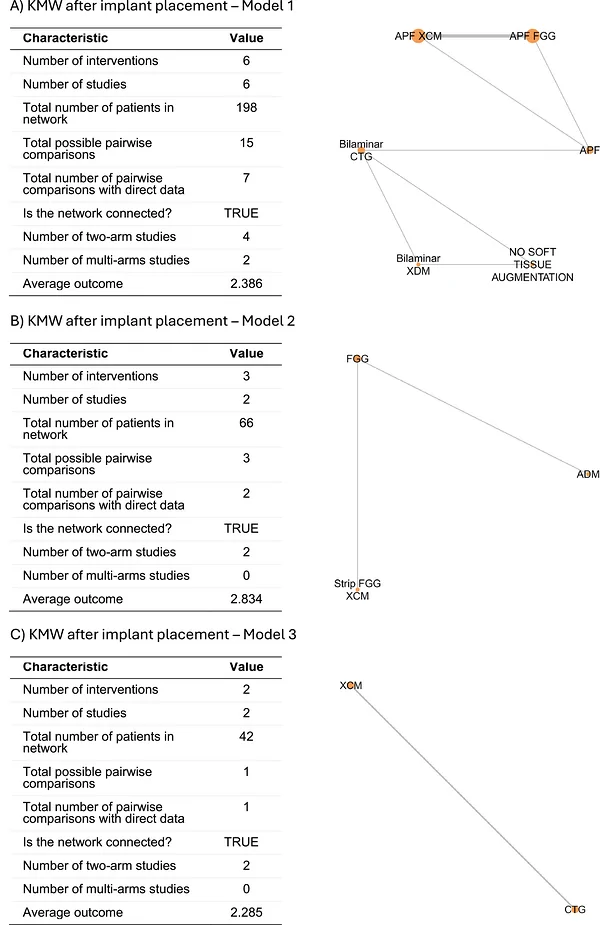
Network meta‐analysis plots for keratinized mucosa width changes.

The synthesis of treatment arms and outcome measures included in the NMA models are depicted in Figures 4 and 5, Tables 1 and 2, and Appendix 2 under “Supporting Information” in the online *Journal of Periodontology*. Both direct and indirect comparisons for the outcomes of interest could be calculated. Of the 40 RCTs included in this systematic review, 28 (70.0%) could be included in five sets of NMA, two regarding MT change and three pertaining to KMW change after STA.

**TABLE 1:** Direct and network pairwise comparisons of different therapies in terms of mucosal thickness change after 6 to 12 months of follow‐up (mean differences).

(A) STA at the time of implant placement					
CTG	0.00 [−0.55; 0.56]	.	**0.68 [0.35; 1.00]**	**0.77 [0.02; 1.52]**	**0.89 [0.13; 1.64]**
0.09 [−0.38; 0.57]	ADM	.	0.50 [−0.11; 1.11]	.	.
0.28 [−0.60; 1.15]	0.18 [−0.76; 1.13]	PRF	0.38 [−0.44; 1.20]	.	.
**0.66 [0.35; 0.97]**	**0.56 [0.08; 1.05]**	0.38 [−0.44; 1.20]	No STA	.	.
**0.77 [0.02; 1.52]**	0.68 [−0.21; 1.57]	0.49 [−0.66; 1.65]	0.11 [−0.70; 0.93]	GBR	.
**0.89 [0.13; 1.64]**	0.79 [−0.10; 1.68]	0.61 [−0.54; 1.76]	0.23 [−0.59; 1.04]	0.12 [−0.95; 1.18]	VXCM

(B) STA after implant placement			
CTG	0.17 [−0.18; 0.52]	**0.30 [0.17; 0.43]**	**0.33 [0.15; 0.51]**
0.17 [−0.18; 0.52]	VXCM	.	.
**0.30 [0.17; 0.43]**	0.13 [−0.24; 0.50]	XCM	.
**0.33 [0.15; 0.51]**	0.16 [−0.23; 0.55]	0.03 [−0.19; 0.25]	CGFG

*Notes*: Treatments are ranked from best to worst along the leading diagonal. Above the leading diagonal are estimates from pairwise meta‐analyses; below the leading diagonal are estimates from network meta‐analyses. Significant differences are marked in bold.

Abbreviations: ADM, allogeneic dermal matrix; CGFG, concentrated growth factor graft; CTG, connective tissue graft; GBR, guided bone regeneration; PRF, platelet‐rich fibrin; STA, soft tissue augmentation; VXCM, volume‐stable xenogeneic collagen matrix; XCM, bilayer xenogeneic collagen matrix.

**TABLE 2:** Direct and network pairwise comparisons of different therapies in terms of keratinized mucosa width change after 6 to 12 months of follow‐up (mean differences).

(A) STA after implant placement – Model 1					
APF+FMG	**1.87 [0.84; 2.91]**	.	.	**3.09 [1.10; 5.08]**	.
**1.87 [0.84; 2.91]**	APF+XCM	.	.	1.13 [−0.82; 3.08]	.
2.48 [−0.98; 5.93]	0.60 [−2.85; 4.05]	Bilaminar XDM	0.25 [−2.00; 2.50]	.	0.89 [−1.14; 2.92]
**2.73 [0.11; 5.34]**	0.85 [−1.76; 3.46]	0.25 [−2.00; 2.50]	Bilaminar CTG	0.32 [−1.59; 2.23]	0.64 [−1.64; 2.92]
**3.05 [1.25; 4.84]**	1.17 [−0.61; 2.95]	0.57 [−2.38; 3.52]	0.32 [−1.59; 2.23]	APF	.
3.37 [−0.11; 6.84]	1.49 [−1.98; 4.96]	0.89 [−1.14; 2.92]	0.64 [−1.64; 2.92]	0.32 [−2.66; 3.30]	No STA

(B) STA after implant placement – Model 2		
FMG	0.23 [−0.17; 0.63]	**0.99 [0.70; 1.28]**
0.23 [−0.17; 0.63]	Strip FMG+XCM	.
**0.99 [0.70; 1.28]**	**0.76 [0.27; 1.25]**	ADM

C) STA after implant placement – Model 3	
CTG	0.18 [−0.25; 0.62]
0.18 [−0.25; 0.62]	XCM

*Notes*: Treatments are ranked from best to worst along the leading diagonal. Above the leading diagonal are estimates from pairwise meta‐analyses; below the leading diagonal are estimates from network meta‐analyses. Significant differences are marked in bold.

Abbreviations: ADM, allogeneic dermal matrix; APF, apically positioned flap; CTG, connective tissue graft; FMG, free mucosal graft; PRF, platelet‐rich fibrin; STA, soft tissue augmentation; XCM, bilayer xenogeneic collagen matrix.

#### 3.5.1 MT change

The results of the NMA pertaining to the treatment estimates for MT changes were separated according to the timing of implant placement (i.e., simultaneous with or after implant placement):

- MT changes following STA at the time of implant placement (Figure 4A):
  This NMA included data from 14 RCTs^27^, ^28^, ^31^, ^33^, ^36^, ^40^, ^41^, ^44^, ^46^, ^47^, ^50^, ^61^, ^62^, ^63^ and six different types of interventions (i.e., no STA [reference group], ADM, CTG, guided bone regeneration [GBR], platelet‐rich fibrin [PRF], and volume‐stable XCM). All treatment arms involving the use of autogenous soft tissue grafts or substitute scaffolds were based on bilaminar techniques. CTG was ranked as the best treatment in the short‐term (Table 1A). Significant differences through direct estimates were found in the comparison between CTG and no STA (0.68; 95%CI 0.35–1.00; *p* < 0.05), CTG and GBR (0.77, 95%CI 0.02–1.52; *p* < 0.05), and CTG and VXCM (0.89, 95%CI 0.13–1.64; *p* < 0.05]. The NMA model identified significant differences for the same comparisons (i.e., CTG vs. no STA, CTG vs. GBR, and CTG vs. volume‐stable XCM). No evidence of heterogeneity and no significant inconsistencies in the tested model were identified (Appendices 2 and 3 in the online *Journal of Periodontology*).
- MT changes after STA following implant placement (Figure 4B):
  This NMA included data from three studies^32^, ^34^, ^45^ and four interventions (i.e., CTG, volume‐stable XCM, bilayer XCM and concentrated growth factors). CTG was ranked as the best treatment in the short‐term (Table 1B). Both direct and indirect comparisons revealed significant differences between CTG and bilayer XCM (0.30; 95%CI 0.17–0.43; *p* < 0.05) and CTG and concentrated growth factors (0.33; 95%CI 0.15–0.51; *p* < 0.05), but the difference was not significant between CTG and volume‐stable XCM (0.17; 95%CI −0.18 to 0.52; *p* < 0.05). Low heterogeneity and no inconsistencies in the tested model could be identified (Appendices 2 and 3).

#### 3.5.2 KMW change

A total of 10 RCTs (22 groups) comprising 11 different treatment arms provided potentially eligible information to be included in NMA models.^29^, ^30^, ^37^, ^43^, ^48^, ^49^, ^53^, ^54^, ^56^, ^59^ However, after thorough data checking, it was identified that the existing therapies comprise three disconnected networks (i.e., the set of therapies was split into three subnetworks, with no subset sharing common treatment arms: Subset 1. Apically positioned flap (APF), APF+FMG, APF+XCM, Bilaminar(CTG), Bilaminar(XCM) and no STA; Subset 2. ADM, FMG and Strip FMG+XCM; and Subset 3. CTG and XCM (Appendix 2). Therefore, it was not possible to combine the 11 different treatment arms as single therapies into a more precise NMA model for the outcome KMW augmentation.

Given the available groups, the following subnetwork meta‐analysis models were constructed, all of them pertaining to STA with the primary purpose of achieving KMW gain after implant placement:

- KMW Model 1 (Figure 5A): This NMA model included data from six trials^29^, ^43^, ^49^, ^53^, ^54^, ^59^ and six different interventions (i.e., APF, APF+FMG, APF+XCM, bilaminar CTG, bilaminar bilayer XCM, and no STA). APF+FMG was ranked as the best treatment in the short‐term (Table 2A). There was evidence of significant differences for direct and NMA comparisons between APF+FMG and APF+XCM (1.87; 95%CI 0.84–2.91; *p* < 0.05), as well as for indirect comparisons between APF+FMG and bilaminar CTG (2.73; 95%CI 0.11–5.34; *p* < 0.05) and between APF+FMG and APF (3.05; 95%CI 1.25–4.84; *p* < 0.05). Moderate to high heterogeneity and no significant inconsistency were identified in the tested model (Appendices 2 and 3).
- KMW Model 2 (Figure 5B): This NMA model included data from two trials^30^, ^37^ and three different interventions (i.e., ADM, FMG, and strip FMG+XCM). Given the nature of the interventions, only non‐submerged STA approaches were included in this analysis. FMG was ranked as the best treatment in the short‐term (Table 2B). Significant differences were identified for direct and indirect comparisons regarding FMG and ADM (0.99; 95%CI 0.70–1.28; *p* < 0.05), as well as for NMA comparing strip FMG+XCM and ADM (0.76; 95%CI 0.27–1.25. *p* < 0.05). Heterogeneity outcomes were not available for this model, and no significant inconsistencies in the tested model could be identified (Appendices 2 and 3).
- KMW Model 3 (Figure 5C): This NMA model included data from two RCTs^48^, ^56^ and two types of interventions (i.e., CTG and bilayer XCM) based on non‐submerged surgical approaches. CTG was ranked as the best treatment in the short‐term (Table 2C), but no significant differences were found among the direct and indirect treatment slopes. No evidence of heterogeneity was detected, and inconsistency outcomes were not available for this model (Appendices 2 and 3).

## 4 DISCUSSION

### 4.1 Summary of main findings

By synthesizing and analyzing relevant evidence extracted from a substantial number of clinical trials (*n* = 40),^27^, ^28^, ^29^, ^30^, ^31^, ^32^, ^33^, ^34^, ^35^, ^36^, ^37^, ^38^, ^39^, ^40^, ^41^, ^42^, ^43^, ^44^, ^45^, ^46^, ^47^, ^48^, ^49^, ^50^, ^51^, ^52^, ^53^, ^54^, ^55^, ^56^, ^57^, ^58^, ^59^, ^60^, ^61^, ^62^, ^63^, ^64^, ^65^, ^66^ this systematic review sought to clarify the relative advantages and limitations of autogenous soft tissue grafts and substitute scaffolds for STA in the context of implant therapy, with the goal of guiding clinicians in selecting the most appropriate technique for optimizing key CROs and PROs/PREs depending on specific clinical scenarios.

Considering the data extracted from the RCTs included in this study, the conduction of a set of quantitative analyses (NMA) pertaining to MT and KMW changes was feasible. These NMAs revealed that regardless of whether STA is performed simultaneously with or after implant placement, a bilaminar approach involving the use of an autogenous CTG stands as the best treatment for MT augmentation in the short term (up to 12 months), as shown in Table 1. Regarding therapeutic options primarily aimed at achieving KMW gain that were performed after implant placement, two NMA models showed that the use of an FMG after creating a vascular bed (APF) is the best treatment, followed by APF with a bilayer XCM. Model 3, which encompassed two studies that compared CTG with XCM,^48^, ^56^ did not identify significant differences between these treatment modalities. These quantitative analyses generally indicate that autogenous grafts, either CTG or FMG, remain the treatments of reference to achieve predictable MT and KMW gains in implant therapy. However, other treatment options involving the use of substitute scaffolds should be considered as viable alternatives given their similar performance, particularly in terms of MT gain. These findings are in alignment with those reported in previously published systematic reviews on this topic.^2^, ^9^, ^13^

A meticulous qualitative assessment of the evidence revealed that CROs and PROs/PREs of interest were inconsistently reported in the literature. The most frequently reported CROs were implant survival rate and esthetic scores (50% of studies), followed by MT changes (45% of studies) and marginal bone level changes (37.5% of studies). Regarding esthetic outcomes, it was observed that while the use of an autogenous CTG rendered superior esthetics when the main treatment objective is to obtain MT and volume gain, when the priority is to increase the band of KMW the use of an FMG is esthetically outperformed by specific substitute scaffolds, in particular bilayer XCM. This observation regarding a superior texture and color integration of this material compared with autogenous FMG is consistent with the findings of previous SRs and clinical studies.^8^, ^13^, ^68^, ^69^ In contrast, the vast majority of studies included in this review showed that patient‐reported esthetic perception did not substantially differ whether an autogenous CTG or a substitute scaffold was employed for STA, as concluded in another systematic review.^15^ Another very relevant finding pertaining to PROs was that patients who underwent STA involving the use an autogenous grafts (CTG or FMG) harvested from an intraoral source consistently reported more discomfort compared with those individuals who received a substitute scaffold. This important observation has been highlighted in another comprehensive review on this topic,^14^ and a large multicenter study involving over 80 patients.^17^ Thus, this should be taken into consideration in clinical decision‐making processes. Notably, postoperative discomfort was assessed in 15 of the 40 selected articles.^12^, ^32^, ^37^, ^39^, ^40^, ^41^, ^43^, ^46^, ^49^, ^53^, ^54^, ^56^, ^58^, ^59^, ^61^ In the majority, this was measured up to 14 days postoperatively, with the exception of two studies that extended this assessment to 30 days after surgery.^56^, ^61^ Methodological assessments were quite consistent and standardized across this subset of selected studies as in almost all of them, a visual analog scale (VAS) from 0 to 10 or 100 was employed. Only in one study was a verbal descriptive scale used to determine the level of pain (from 0 to 10) at 1 and 5 days postoperatively.^59^

### 4.2 Limitations and potential biases in the review process

The results of the RoB analysis indicate that the overall quality of clinical evidence available in the selected literature is high since only three of the included studies exhibited a high risk of bias (Figure 2).^29^, ^48^, ^63^

As noted in the previous section, most outcomes of interest were not frequently nor consistently reported in the included RCTs. Among the most relevant CROs, data on changes in STH and marginal mucosa position over time were particularly scarce. This prevented us from conducting more robust and diverse quantitative analyses to shed light on the performance of different surgical treatment modalities of STA for peri‐implant phenotype modification from various angles, including possible relationships or associations between key CROs and PROs/PREs. Furthermore, when interpreting the results of the NMA conducted in this systematic review, it should be taken into consideration that this information pertains to the short‐term (i.e., ≤12 months). Although a few of the trials included in this review reported findings observed after 3 or 5 years of follow‐up,^31^, ^58^, ^61^, ^66^ the vast majority of studies from which data to conduct the NMA were extracted did not extend their final follow‐up beyond 1 year. This highlights the need for well‐designed clinical trials with long‐term follow‐ups (≥5 years) to determine the longevity and reliability of CROs and PROs after STA therapies involving the use of different materials (autogenous grafts vs. substitute scaffolds) and guide clinical decision‐making processes in daily practice.^1^

The influence of the modality of placement (i.e., immediate vs. delayed) on key outcomes of interest related to STA could not be assessed through a quantitative analysis due to the absence of direct comparisons in the selected literature and the limited data on key outcomes of interest, particularly KMW and STH changes, across different studies involving either placement modality. This was also noted in a previously published systematic review.^70^ However, a critical analysis of the results associated with either placement option is not indicative of clinically meaningful differences between them, suggesting that whether implants are placed in an immediate or delayed fashion should not affect the results of STA, as long as these interventions are properly performed. However, this observation should be taken with caution, given the paucity of data derived from direct comparative studies.

Although some of the NMAs could have been stratified as a function of the timing of STA (simultaneous with or after implant placement) and the follow‐up period (i.e., 6 to 12 months after treatment), other factors that may have impacted the pooled estimates could not be incorporated into the main analyses.

For instance, while both rankings for mucosa thickness changes (from best to worst) included only bilaminar procedures, the NMA for KTW changes involved different monolaminar and STA approaches that could not be stratified into more specific analyses. Furthermore, it was not possible to assess differences in baseline soft tissue phenotype among sites that did or did not undergo STA, or between sites treated with autogenous grafts versus substitute scaffolds. Additionally, whether the outcomes observed after specific therapeutic approaches differ between single, tooth‐bound and multiple, contiguous implant sites, and if inter‐implant distance is a relevant variable, could not be explored in this study given the paucity of data in the selected articles.

It should be noted that, in order to overcome some of the limitations of substitute scaffolds in obtaining KMW, MT, STH, and marginal mucosa position changes comparable or superior to those associated with the use of autogenous grafts, combining them with biologics may be a plausible strategy. However, no relevant evidence regarding this combination therapeutic approach was identified in this systematic review. Similarly, evidence from RCTs reporting on medium‐ to long‐term follow‐ups suggests that STA may provide more stable mucosal margin outcomes, a pivotal outcome in clinical practice. These observations should be considered in future research.

In addition, it can be argued that both NMA models evaluating mucosa width changes and Models 2 and 3 (pertaining to MT changes analysis) identified no or low heterogeneity and no inconsistencies (Appendices 2 and 3). These findings suggest that the NMA model applied is a good fit for the data and that the results can be trusted. Regarding Model 1 for testing MT changes, moderate to high heterogeneity was detected, but no significant inconsistency was found. This suggests that, while there is significant variation among studies, direct and indirect evidence seem to remain in agreement. Conversely, the limited number of studies and the variety of surgical procedures reported should be considered when interpreting these results.

### 4.3 Agreements or disagreements with other studies or reviews

Only in the past 5 years, numerous systematic reviews on the topic of STA in the context of implant therapy have been published in high impact factor journals in the field of dentistry.^1^, ^2^, ^8^, ^9^, ^13^, ^14^, ^15^, ^23^, ^24^, ^71^, ^72^, ^73^ The main findings of these reviews are generally consistent among them and also in agreement with the findings hereby reported. In summary, high‐level evidence suggests that STA aimed at enhancing the phenotypical features of peri‐implant soft tissues (particularly, KMW and MT) is effective and beneficial, contributing to optimal peri‐implant health and esthetic outcomes, and preventing the occurrence of biological complications (e.g., peri‐implant mucositis, peri‐implantitis, and soft tissue dehiscences). Notably, this contrasts with one of the conclusions of the S3 level clinical practice guideline on “Prevention and treatment of peri‐implant diseases” that indicates that the effect of STA on peri‐implant health is unclear.^74^

However, it should be kept in mind that the status of the peri‐implant tissues, both bone and soft tissue, is only one of the keys that may determine the success of tooth replacement therapy with implant‐supported prostheses. Other important aspects that should be taken into consideration in the continuum of care are an appropriate control of systemic risk factors, patient education, including behavioral changes, if necessary (e.g., smoking cessation), adequate implant position and prosthetic design,^75^ and personalized maintenance programs that contribute to long‐term peri‐implant tissue stability and quality of life.

## 5 CONCLUSIONS

Considering the findings of this systematic review, the following conclusions may be posed:

- Bilaminar techniques involving the use of autogenous connective tissue grafts performed either simultaneously with or after implant placement represent the gold standard to achieve mucosal thickness gains.
- The application of a free mucosal graft (FMG) over a partial‐thickness vascular bed performed after implant placement represents the gold standard for obtaining keratinized mucosa width gains.
- Treatment options involving the use of substitute scaffolds may be considered as viable alternatives given their similar performance compared with autogenous soft tissue grafts, particularly in terms of MT gain. However, it must be acknowledged that the superiority of connective tissue grafts in terms of volume gains is consistent and may carry long‐term benefits not fully captured in the relatively short follow‐up range of many studies included in this review.
- While the use of an autogenous CTG typically renders superior esthetics compared with substitute scaffolds when the primary therapeutic goal is to obtain MT and volume gain, when the priority is to augment the band of KMW, the use of an FMG is esthetically outperformed by bilayer xenogeneic collagen matrices.
- Patient‐reported esthetic perception does not substantially differ whether an autogenous CTG or a substitute scaffold is employed for soft tissue augmentation.
- The use of substitute scaffolds is associated with lower postoperative morbidity compared with autogenous soft tissue grafts.
- The impact of using autogenous soft tissue grafts versus substitute scaffolds for soft tissue augmentation, in relation to the timing of augmentation and the implant placement approach, could not be established owing to the limited number of studies available for quantitative analysis.

## 6 IMPLICATIONS AND RECOMMENDATIONS FOR FUTURE RESEARCH

Well‐conducted and appropriately sized RCTs aimed at assessing and analyzing meaningful long‐term CROs and PROs/PREs comparing different modalities of STA with each other and with other therapies are warranted to inform decision‐making processes in clinical practice. The performance of the combined use of biologics (e.g., growth factors or pluripotent mesenchymal stem cells) with substitute scaffolds versus autogenous soft tissue grafts should be tested in future clinical trials. Also, studies that directly compare the outcomes of STA as a function of implant placement modality (immediate vs. delayed) should be performed to determine whether this technical aspect plays an important role. In accordance with the Implant Dentistry Core Outcome Set and Measurement (ID‐COSM) recommendations,^22^ future studies should incorporate composite outcomes to comprehensively assess the performance of different surgical modalities of STA, including linear (e.g., changes in KMW, MT, STH, and marginal mucosa position), profilometric, and volumetric assessments, as well as relevant PROs/PREs, exploring possible associations with CROs of interest. These outcomes should be assessed with standardized and reliable tools and recorded at different time points, including long‐term follow‐ups, to allow for meaningful quantitative analyses in the future.

## AUTHOR CONTRIBUTIONS

**Gustavo Avila‐Ortiz**: conceived the scope and initial structure of the systematic review; verified the validity and standardization of collected data; led the writing. **Leandro Chambrone**: conceived the scope and initial structure of the systematic review; performed the electronic search; performed the data analysis; led the writing. **Ronald Jung**: conceived the scope and initial structure of the systematic review; critically revised the final version of the manuscript. **Miha Pirc**: performed the screening and selected the articles; assessed the risk of bias; critically revised the final version of the manuscript. **Franz J. Strauss**: performed the screening and selected the articles; assessed the risk of bias; critically revised the final version of the manuscript. **Daniel Thoma**: verified the final article selection according to protocol; critically revised the final version of the manuscript. **Sandra Stuhr**: extracted the data; critically revised the final version of the manuscript. **Emilio Couso‐Queiruga**: extracted the data; verified the risk of bias assessment; led the writing.

## CONFLICT OF INTEREST STATEMENT

The authors have no conflicts of interest to report pertaining to the conduction of this systematic review.

No financial support or sponsorship was received.

## Supporting information



Supporting Information

Supporting Information

Supporting Information

Supporting Information
