# Supplementary material for: Autogenous grafts versus substitute scaffolds for soft tissue augmentation in implant therapy: A systematic review
Source: J Periodontol. 2026 Sep 14;97(Suppl 1):S52–69. doi: 10.1002/jper.70104 (PMC13575472; doi:10.1002/jper.70104)
Supplement: Supplementary file 1 [file JPER-97-S52-s001.docx]

**Appendix 2. Assessment of inconsistency for all studies included into NMA**

**A) Mucosa thickness change at the time of implant placement**

| **Comparison** | **No. Studies** | **NMA** | **Direct** | **Indirect** | **Difference** | **Diff. 95CI lower** | **Diff. 95CI upper** | **P Value** |
| --- | --- | --- | --- | --- | --- | --- | --- | --- |
| ADM:CTG | 2 | -0.09 | -0.00 | -0.34 | 0.34 | -0.73 | 1.40 | 0.54 |
| ADM:GBR | 0 | 0.68 | NA | 0.68 | NA | NA | NA | NA |
| ADM:NO_SOFT_TISSUE_AUGMENTATION | 2 | 0.56 | 0.50 | 0.67 | -0.16 | -1.17 | 0.84 | 0.75 |
| ADM:PRF | 0 | 0.18 | NA | 0.18 | NA | NA | NA | NA |
| ADM:VXCM | 0 | 0.79 | NA | 0.79 | NA | NA | NA | NA |
| CTG:GBR | 2 | 0.77 | 0.77 | NA | NA | NA | NA | NA |
| CTG:NO_SOFT_TISSUE_AUGMENTATION | 7 | 0.66 | 0.68 | 0.39 | 0.29 | -0.97 | 1.54 | 0.65 |
| CTG:PRF | 0 | 0.28 | NA | 0.28 | NA | NA | NA | NA |
| CTG:VXCM | 2 | 0.89 | 0.89 | NA | NA | NA | NA | NA |
| GBR:NO_SOFT_TISSUE_AUGMENTATION | 0 | -0.11 | NA | -0.11 | NA | NA | NA | NA |
| GBR:PRF | 0 | -0.49 | NA | -0.49 | NA | NA | NA | NA |
| GBR:VXCM | 0 | 0.12 | NA | 0.12 | NA | NA | NA | NA |
| PRF:NO_SOFT_TISSUE_AUGMENTATION | 1 | 0.38 | 0.38 | NA | NA | NA | NA | NA |
| VXCM:NO_SOFT_TISSUE_AUGMENTATION | 0 | -0.23 | NA | -0.23 | NA | NA | NA | NA |
| PRF:VXCM | 0 | 0.61 | NA | 0.61 | NA | NA | NA | NA |

**B) Mucosa thickness change after implant placement**

| **Comparison** | **No. Studies** | **NMA** | **Direct** | **Indirect** | **Difference** | **Diff. 95CI lower** | **Diff. 95CI upper** | **P Value** |
| --- | --- | --- | --- | --- | --- | --- | --- | --- |
| Concentrated_growth_factor_graft:CTG | 1 | -0.33 | -0.33 | NA | NA | NA | NA | NA |
| Concentrated_growth_factor_graft: VXCM | 0 | -0.14 | NA | -0.14 | NA | NA | NA | NA |
| Concentrated_growth_factor_graft:XCM | 0 | -0.03 | NA | -0.03 | NA | NA | NA | NA |
| CTG:VXCM | 2 | 0.19 | 0.19 | NA | NA | NA | NA | NA |
| CTG:XCM | 1 | 0.30 | 0.30 | NA | NA | NA | NA | NA |
| VXCM:XCM | 0 | 0.11 | NA | 0.11 | NA | NA | NA | NA |

**C) Keratinized mucosa width change after implant placement – model 1**

| **Comparison** | **No.Studies** | **NMA** | **Direct** | **Indirect** | **Difference** | **Diff_95CI_lower** | **Diff_95CI_upper** | **P Value** |
| --- | --- | --- | --- | --- | --- | --- | --- | --- |
| APF_FMG:APF | 1 | 3.05 | 3.09 | 2.86 | 0.23 | -4.31 | 4.77 | 0.92 |
| APF_XCM:APF | 1 | 1.17 | 1.13 | 1.37 | -0.24 | -4.96 | 4.47 | 0.92 |
| Bilaminar_CTG_:APF | 1 | 0.32 | 0.32 | NA | NA | NA | NA | NA |
| Bilaminar_XDM_:APF | 0 | 0.57 | NA | 0.57 | NA | NA | NA | NA |
| NO_SOFT_TISSUE_AUGMENTATION:APF | 0 | -0.32 | NA | -0.32 | NA | NA | NA | NA |
| APF_FMG:APF_XCM | 4 | 1.87 | 1.87 | NA | NA | NA | NA | NA |
| APF_FMG:Bilaminar_CTG_ | 0 | 2.73 | NA | 2.73 | NA | NA | NA | NA |
| APF_FMG:Bilaminar_XDM_ | 0 | 2.48 | NA | 2.48 | NA | NA | NA | NA |
| APF_FMG:NO_SOFT_TISSUE_ AUGMENTATION | 0 | 3.37 | NA | 3.37 | NA | NA | NA | NA |
| APF_XCM:Bilaminar_CTG_ | 0 | 0.85 | NA | 0.85 | NA | NA | NA | NA |
| APF_XCM:Bilaminar_XDM_ | 0 | 0.60 | NA | 0.60 | NA | NA | NA | NA |
| APF_XCM:NO_SOFT_TISSUE_AUGMENTATION | 0 | 1.49 | NA | 1.49 | NA | NA | NA | NA |
| Bilaminar_CTG_:Bilaminar_XDM_ | 1 | -0.25 | -0.25 | NA | NA | NA | NA | NA |
| Bilaminar_CTG_:NO_SOFT_TISSUE_AUGMENTATION | 1 | 0.64 | 0.64 | NA | NA | NA | NA | NA |
| Bilaminar_XDM_:NO_SOFT_TISSUE_AUGMENTATION | 1 | 0.89 | 0.89 | NA | NA | NA | NA | NA |

**D) Keratinized mucosa width change after implant placement – model 2**

| **Comparison** | **No.Studies** | **NMA** | **Direct** | **Indirect** | **Difference** | **Diff_95CI_lower** | **Diff_95CI_upper** | **pValue** |
| --- | --- | --- | --- | --- | --- | --- | --- | --- |
| ADM:FMG | 1 | -0.99 | -0.99 | NA | NA | NA | NA | NA |
| ADM:Strip_FMG_XCM | 0 | -0.76 | NA | -0.76 | NA | NA | NA | NA |
| Strip_FMG_XCM:FMG | 1 | -0.23 | -0.23 | NA | NA | NA | NA | NA |

**E) Keratinized mucosa width change after implant placement – model 3**

| **Comparison** | **No.Studies** | **NMA** | **Direct** | **Indirect** | **Difference** | **Diff_95CI_lower** | **Diff_95CI_upper** | **pValue** |
| --- | --- | --- | --- | --- | --- | --- | --- | --- |
| XCM:CTG | 2 | -0.18 | -0.18 | NA | NA | NA | NA | NA |
