# Supplementary material for: Autogenous grafts versus substitute scaffolds for soft tissue augmentation in implant therapy: A systematic review
Source: J Periodontol. 2026 Sep 14;97(Suppl 1):S52–69. doi: 10.1002/jper.70104 (PMC13575472; doi:10.1002/jper.70104)
Supplement: Supplementary file 2 [file JPER-97-S52-s003.docx]

|  | Main reason for exclusion |
| --- | --- |
| Akcali et al. 2014 | No intervention of interest |
| Al-Diasty et al. 2022 | Insufficient sample size |
| Anderson et al. 2014 | Unclear or insufficient follow-up time |
| Ashurko et al. 2022 | Unclear or insufficient follow-up time |
| Azaripour et al. 2021 | Concerns about data reliability  (These two publications are very similar) |
| Azaripour et al. 2023 |  |
| Bianchi et al. 2024 | Unclear or no randomization |
| Buyukozdemir et al. 2015 | Insufficient sample size |
| Byun et al. 2020 | No intervention of interest |
| Cardaropoli et al. 2014 | No intervention of interest |
| Cosyn et al. 2021 | Unclear or insufficient follow-up time |
| Cosyn et al. 2022 | Same cohort as in Surdiacourt et al. 2024 |
| De Angelis et al. 2021 | Unclear or no randomization |
| De Angelis et al. 2023 | Unclear or no randomization |
| De Bruyckere et al. 2018 | Same cohort as in Bouckaert et al. 2022 |
| Donker et al. 2024 | No intervention of interest |
| Eeckhout et al. 2023 | Same cohort as in Cosyn et al. 2024 |
| Esfahani et al. 2022 | No outcomes of interest reported |
| Farina & Zaffe 2015 | Unclear or insufficient follow-up time |
| Frizzera et al. 2019 | Insufficient sample size |
| Froum et al. 2015 | Unclear or insufficient follow-up time |
| Fujita et al. 2021 | Unclear or no randomization |
| Hadzik et al. 2023 | Unclear methodology / stud design |
| Hamdy et al. 2021 | No intervention of interest |
| Hammerle et al. 2023 | Unclear or insufficient follow-up time |
| Happe et al. 2021 | Concerns about data reliability  (These two publications are very similar) |
| Happe et al. 2022 |  |
| Hof et al. 2014 | Unclear or no randomization |
| Huber et al. 2017 | Same cohort as in Thoma et al. 2023 |
| Hutton et al. 2018 | Unclear or insufficient follow-up time |
| Kanafi et al. 2023 | Unclear or insufficient follow-up time |
| Kotsailidi et al. 2022 | Unclear or no randomization |
| Momeni et al. 2024 | Insufficient sample size |
| Naiem et al. 2023 | Insufficient sample size |
| Namadmalian et al. 2021 | Unclear or insufficient follow-up time |
| Oh et al. 2017 | Inclusion of peri-implantitis patients |
| Oh et al. 2020 |  |
| Papapetros et al. 2019 | Unclear or insufficient follow-up time |
| Patnaik et al. 2023 | Unclear or insufficient follow-up time |
| Poli et al. 2019 | Insufficient sample size |
| Puzio et al. 2018 | Unclear methodology / stud design |
| Puzio et al. 2020 | Same cohort as in Hadzik et al. 2023 |
| Ramanauskaite et al. 2023 | Correction of another article |
| Rojo et al. 2018 | Unclear or insufficient follow-up time |
| Rokn et al. 2020 | Insufficient sample size |
| Rungcharassaeng et al. 2012 | Unclear or no randomization |
| Schallhorn et al. 2015 | Unclear or no randomization |
| Schmitt et al. 2012 | Insufficient sample size |
| Schmitt et al. 2020 | Insufficient sample size |
| Stimmelmayer et al. 2011 | Unclear or no randomization |
| Tabanella & Viale 2024 | No outcomes of interest reported |
| Thoma et al. 2016 | Unclear or insufficient follow-up time |
| Thoma et al. 2018 | Unclear or insufficient follow-up time |
| Thoma et al. 2020 | Same cohort as in Thoma et al. 2023 |
| Ustaoglu et al. 2020 | Unclear or insufficient follow-up time |
| Vellis et al. 2019 | Unclear or no randomization |
| Verardi et al. 2019 | Unclear or no randomization |
| Wang et al. 2024 | Unclear or insufficient follow-up time |
| Zafiropoulos et al. 2016 | Insufficient sample size |
| Zang et al. 2022 | Unclear or insufficient follow-up time |
| Zeltner et al. 2017 | Unclear or insufficient follow-up time |
| Zheng et al. 2021 | No intervention of interest |
| Zuercher et al. 2023 | Insufficient sample size |
| Zuiderveld et al. 2018 | Same cohort as in Zuiderveld et al. 2024 |
| Zuiderveld et al. 2020 | Same cohort as in Zuiderveld et al. 2024 |
