# Supplementary material for: Autogenous grafts versus substitute scaffolds for soft tissue augmentation in implant therapy: A systematic review
Source: J Periodontol. 2026 Sep 14;97(Suppl 1):S52–69. doi: 10.1002/jper.70104 (PMC13575472; doi:10.1002/jper.70104)
Supplement: Supplementary file 3 [file JPER-97-S52-s004.docx]

**Appendix 3. Forest plots and respective ‘tau’ values (i.e. the between-study standard deviation, representing the amount of heterogeneity among the true effect sizes of the studies).**

**A) Mucosa thickness change at the time of implant placement**

Between-study standard deviation (tau): 0.39

**B) Mucosa thickness change after implant placement**

Between-study standard deviation (tau): 0

**C) Keratinized mucosa width change after implant placement – model 1**

Between-study standard deviation (tau): 0.97

**D) Keratinized mucosa width change after implant placement – model 2**

Between-study standard deviation (tau): NA

**E) Keratinized mucosa width change after implant placement – model 3**

Between-study standard deviation (tau): 0
